# Transcriptome and phytohormone analysis reveals a comprehensive phytohormone and pathogen defence response in pear self-/cross-pollination

**DOI:** 10.1007/s00299-017-2194-0

**Published:** 2017-09-08

**Authors:** Dongqing Shi, Chao Tang, Runze Wang, Chao Gu, Xiao Wu, Shi Hu, Jin Jiao, Shaoling Zhang

**Affiliations:** Centre of Pear Engineering Technology Research, State Key Laboratory of Crop Genetics and Germplasm Enhancement, Nanjing Agricultural University, Nanjing, 210095 China

**Keywords:** Pear (*Pyrus bretschneideri Rehd.*), Self-incompatibility, Transcriptome, S-RNase, Methyl jasmonate

## Abstract

**Key message:**

**Candidate genes were identified and the role of phytohormones such as JA-Me and ABA in the synthesis of S-RNase was emphasized in pear self-incompatibility.**

**Abstract:**

Self-incompatibility (SI) occurs widely in flowering plants as an intraspecific reproductive barrier. This phenomenon promotes variation within species, but for some species such as *Pyrus*, SI is a nuisance rather than a benefit in agricultural production. Although many studies have been conducted on SI in pears, its mechanism remains unclear. In this study, high-throughput Illumina RNA sequencing (RNA-seq) was used to identify SI-related genes in pear styles. Using transcriptome comparisons, differentially expressed genes of unpollinated (UP), cross-pollinated (CP), and self-pollinated (SP) styles were identified after 48 h. A total of 1796 and 1890 genes were identified in DSC (UP vs. CP) and DSI (UP vs. SP), respectively. KEGG analysis revealed that genes involved in the “plant hormone signal transduction pathway” and “plant–pathogen interaction pathway” were significantly enriched in DSI (UP vs. SP) compared to those in DSC (UP vs. CP). The expression level of S-glycoprotein ribonuclease (S-RNase) was dramatically reduced in cross-pollinated (CP) styles. To better understand the relationship between the expression patterns of S-RNase and two major KEGG pathways, the concentrations of phytohormones were measured, and the expression pattern of S-RNase was analysed using qRT-PCR. Our results demonstrate that methyl jasmonate and abscisic acid may enhance the expression level of S-RNase, and pollination can affect the synthesis of methyl jasmonate and abscisic acid in pear styles. Overall, this study is a global transcriptome analysis of SI in pear. A relationship between self-rejection, plant hormones, and pathogen defence was shown in pear.

**Electronic supplementary material:**

The online version of this article (doi:10.1007/s00299-017-2194-0) contains supplementary material, which is available to authorized users.

## Introduction

Most flowering plants with hermaphrodite flowers have evolved a strategy to prevent self-pollination and promote outcrossing. A ubiquitous strategy is self-incompatibility (SI), which inhibits the growth of self-pollen and accepts the non-self for fertilization (de Nettancourt 1997). Decades of research in biochemistry and molecular biology have gradually uncovered the mechanism of SI (McClure 2004; Nasrallah and Nasrallah 2014; Wilkins et al. 2015). However, the mechanism of pollen tube growth and style recognition remain unclear.

There are two major types of SI: sporophytic SI (SSI), found in Brassicaceae, and gametophytic SI (GSI), found in Rosaceae and Solanaceae. SSI is controlled by the interaction between stigma-specific S-receptor kinase and a pollen-specific S-locus cysteine-rich protein, which inhibits self-pollen only on the surface of the stigma (Indriolo et al. 2014). By contrast, during GSI in Rosaceae and Solanaceae, the pollen tube penetrates into the base of the style, and S-glycoprotein ribonuclease (S-RNase) activity and the pollen-specific S-locus F-box protein (SLF/SFB) determine cross-fertilization and self-inhibition of growth (Liu et al. 2014). Furthermore, pollen–pistil recognition begins in the stigma. This reaction continues until the pollen tube reaches the base of the style in most GSI systems. The GSI mechanism in Papaveraceae involves style-specific ligands that bind to receptors on the membrane of the incompatible pollen tube and trigger a Ca^2+^ signalling cascade, which results in programmed cell death (PCD) of the self-pollen (Takayama and Isogai 2005). Different SI types exhibit different biological processes in space and time.

Pollen tube growth in the style is hyponastic growth during which there is a continuous exchange of signals between the pollen tube and style. RNA degradation is triggered, and a signalling cascade results in PCD culminated by factors that determine style incompatibility (Eaves et al. 2014; Wilkins et al. 2014). Proteins that contribute to reinforcement and secreted proteins, including potential signalling components, are significantly enriched during pollination (Tung et al. 2005). Stigma/stylar cysteine-rich adhesion proteins, chemocyanin, transmitting tissue-specific proteins (TTS), and γ -amino butyric acid (GABA) have been identified in directing pollen tube growth (Swanson et al. 2004). Intriguingly, TTS binds to S-RNase in *Nicotiana* (Hancock et al. 2003). In GSI, the compatible pollen tube in the style sends a signal to reduce the S-RNase level (Liu et al. 2009), which suggests reciprocal signalling crosstalk between the pollen tube and style cells of the transmitting tract.

Overall, SI is a biological process that is controlled by multiple genes and a reciprocal interaction between pollen and the style. High-throughput Illumina RNA sequencing (RNA-seq) is a powerful method to investigate gene regulation and the molecular basis of SI. Candidate genes contributing to SI interaction have been successfully identified in *Leymus chinensis* (Zhou et al. 2014), *Citrus limon* (Zhang et al. 2015), *Solanum* (Zhao et al. 2015), and *Camellia sinensis* (Zhang et al. 2016).

Pear, a perennial and self-incompatible species in the Rosaceae family, is an ancient fruit crop that is cultivated worldwide (Wu et al. 2013). There are large-scale plantings of pear in China. Owing to the lack of self-pollination, considerable time and effort are required every year for the collection of large amounts of pollen and the pollination of trees using pollen consisting of different S alleles. This results in an obvious wastage of land and is not only time-consuming but also causes problems in managing different cultivars. It would be beneficial to farmers if SI could be prevented. S-RNase, is a basic protein with a PI of 9.3–10.0 and a molecular mass of 30 kDa, plays a key role in the inhibition of self-pollen through RNA degradation activity (Hiratsuka et al. 2001). Several studies have been conducted to clarify the mechanism of S-RNase cytotoxicity in incompatible pollen in pear. For example, S-RNase has been reported to trigger mitochondrial alteration and to decrease NADPH oxidase activity in the mitochondria and cytosol (Wang et al. 2010). As a result, tip-localized ROS were disrupted, the nuclear DNA was degraded (Wang et al. 2009), the balance of intracellular Ca^2+^ was altered, and the stability of the actin cytoskeleton was broken (Liu et al. 2007). However, a little work has been conducted on the synthesis and regulation of S-RNase in pear styles. S-RNase activity is necessary for the rejection of incompatible pollen (Huang et al. 1994), and a minimum concentration of S-RNase is also required in the style (Qin et al. 2006). Thus, inhibition of S-RNase activity or a reduction in S-RNase levels in styles may be a good method to prevent the rejection of self-pollen.

In this study, RNA-seq was used to reveal the gene regulation of self- and cross-pollination in pear to better understand SI. Phytohormone analysis and phytohormone treatment combined with RT-PCR were used to establish the relationship between phytohormones and SI in *Pyrus*.

## Materials and methods

### Plant material

Adult pear trees were planted in the orchards of Nanjing Agricultural University, Jangsu, China. The anthers were detached from ‘Dangshan’ pear trees before anthesis. Then, the styles were pollinated with self-pollen (‘Dangshan’) and cross-pollen (‘Cuiguan’). Pollination bags were used to ensure that the styles were not subjected to other types of interference. After 24 and 48 h pollination, unpollinated, self-pollinated, and cross-pollinated styles with stigma (containing pollen or not) were collected, immediately frozen in liquid nitrogen, and stored at −80 °C.

Anthers were detached first; then, the styles were soaked in JA-Me (Sigma, St. Louis, MO, USA), ABA (RYON, Shanghai, China), GA3 (Genview, IL, USA), IAA (Genview, IL, USA), BR (Sigma, St. Louis, MO, USA), and ZR (COMIN, Shanghai, China) for 3 min at the final concentrations of 10, 50, 50, 200, 5, and 100 µM, respectively. Subsequently, pollination bags were used to ensure that the styles were not subjected to other types of interference. All hormones were added to 1% ethanol containing 0.1% Tween 20 solution. This solution was used as the mock control. After 0, 4, 12, 24, and 48 h exogenous treatment, UP styles with stigma were collected, immediately frozen in liquid nitrogen, and stored at −80 °C.

### RNA isolation and Illumina sequencing

The RNAprep Pure Plant Kit (Tiangen, Beijing, China) was used to isolate total RNA according to the manufacturer’s protocol. RNA degradation and contamination were detected on 1% agarose gels. The RNA concentration of these samples was measured using a NanoDrop 2000 (Thermo Scientific, Waltham, MA, USA). The quality was assessed using an Agilent 2100 Bioanalyzer (Agilent Technologies, CA, USA). Only samples with RIN (RNA integrity number) ≥8 and 28S:18SRNA ≥1.5 were used for deep sequencing. The cDNA libraries were prepared from different style samples after 48 h using a TruSeq™ RNA Sample Prep Kit (Illumina, Carlsbad, CA, USA) following the manufacturer’s protocol. Briefly, poly(A) mRNA was enriched using poly-T oligoattached magnetic beads (Invitrogen, Foster City, USA) and was then fragmented into short pieces using an RNA Fragmentation Kit (Ambion, Austin, TX, USA). Double-stranded cDNA was reverse transcribed and amplified. An Illumina HiSeq™ 4000 instrument was used to perform the sequencing reactions.

### HiSeq data analysis

Raw reads containing by the HT-2500 were pass-filtered to exclude reads that contain adapters, low-quality, and unknown bases. The remaining high-quality sequences (clean reads) were mapped to the *Pyrus bretschneideri* genome data (Wu et al. 2013) (http://www.peargenome.njau.edu.cn) using Tophat (Trapnell et al. 2012), permitting no more than five base mismatches in the alignment. The mapped reads were counted using HT-Seq (Anders et al. 2014).

### Identification of DEGs

Quantification of transcript expression was performed using the Reads Per kb per Million reads (RPKM) method (Mortazavi et al. 2008). Differentially expressed genes (DEGs) analysis was performed using the method described by Audic (Audic and Claverie 1997). False discovery rate (FDR) was used to determine the *P* value thresholds in multiple testing. In the statistical analysis, *P* values of <0.05 and fold change ≥2 were marked significantly different in DSI (UP vs. SP) group, DSC (UP vs. CP) group and X (CP vs. SP) groups.

### GO analysis

The Blast2Go program (Conesa et al. 2005) was used to obtain GO annotations for all identified genes. To gain an understanding of the distribution of gene functions at the macro level, the WEGO online tool (Ye et al. 2006) was used to perform GO functional classification. The broad molecular function of the DEGs was analysed in terms of significantly enriched GO categories for molecular function using SEA (Du et al. 2010), in which FDR was calculated to correct the *P* value (Pawitan et al. 2005). *P* values <0.05 and FDRs <0.05 were defined as significant.

### Pathway analysis

KEGG is a highly integrated database for systematic analysis of gene function in terms of the networks of genes and molecules (http://www.genome.jp/kegg/). KEGG pathway analysis was used to identify the significant pathways associated with the DEGs. *P* values <0.05 and FDRs <0.05 were defined as significant (Kanehisa et al. 2008; Yi et al. 2006).

### qRT-PCR analysis

TransScript One-Step gDNA Removal and cDNA Synthesis SuperMix kit (TransGen, Beijing, China) was used to synthesize the first strand cDNA, according to the manufacturer’s instructions. TransStart Tip Green qPCR SuperMix (TransGen, Beijing, China) and the Roche LightCycler 480IIPCR system (Roche Diagnostics GmbH, USA) were used to perform the qRT-PCR. After a total volume of 20 µl reaction mixture was prepared, amplification was carried out with the following cycling parameters: 95 °C for 30 s, followed by 45 cycles of 95 °C for 5 s, and 60 °C for 30 s. Three biological replicates were analysed and the expression values were normalized against actin. The specificity of the products was confirmed via agarose gel and melting curves were analysed. Analysis of the relative gene expression data was conducted using the 2^−∆∆Ct^ method (Livak and Schmittgen 2001).

### Quantification of hormones

Methyl jasmonate (JA), abscisic acid (ABA), gibberellin (GA3), indole-3-acetic acid (IAA), brassinolide (BR), and zeatin riboside (ZR) concentrations were determined using enzyme-linked immunosorbent assay (ELISA) methods according to Yang et al. (2001). The results are presented as the mean ± SE of three replicates.

## Results

### Pear style transcriptome assembly and differential gene expression profiles of DSI (UP vs. SP) and DSC (UP vs. CP)

A total of 65.27, 68.42, and 58.06 M raw reads with an average Q20 over 97% were generated from the UP, SP, and CP samples, respectively, with Illumina Solexa sequencing technology. After removing adaptors, and filtering short and low-quality reads, clean reads of 150 nt were mapped to the reference genome. Of these high-quality reads, 72.2, 72.5, and 70.2% were aligned to the pear reference database (Table 1), and a total of 26,521, 26,982, and 26,882 unigenes were obtained for the UP, SP, and CP samples, respectively.

**Table 1:** Statistics of the reads from RNA-seq analysis and mapping results

Sample	Total clean reads	Single length (bp)	Total length (bp)	GC percentage	Q20 (%)	Mapping to genome rate (%)
Sample UP	64,481,698	150	9,173,193,132	46.03	97.64	72.2
Sample SP	67,636,348	150	9,640,868,615	45.92	97.81	72.5
Sample CP	57,250,730	150	8,125,868,717	45.95	97.71	70.2

**Fig. 1:**
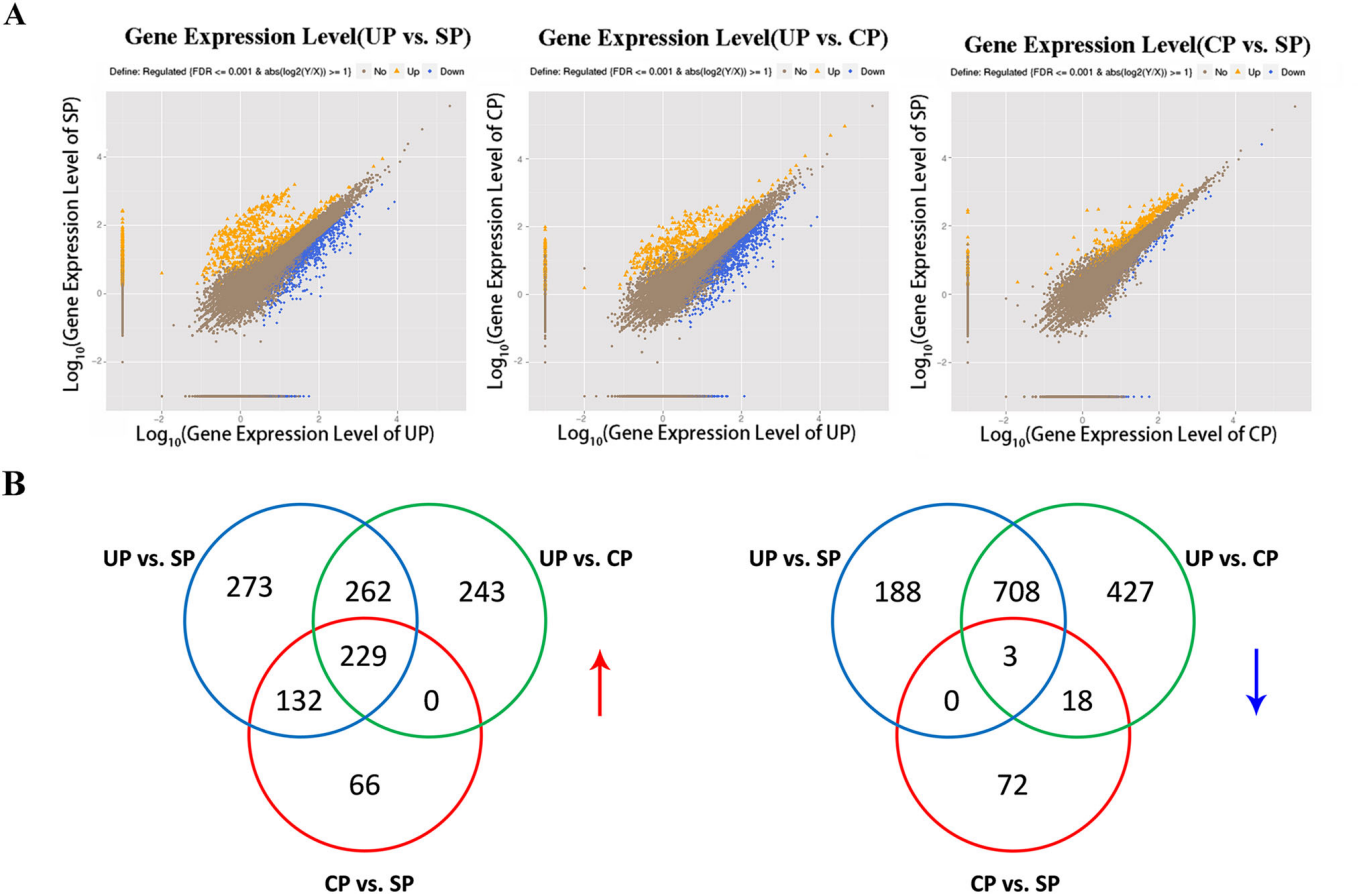
Differentially expressed genes (DEGs) in DSI (UP vs. SP), DSC (UP vs. CP), and X (CP vs. SP). **a** Abundance of each gene was normalized as reads per kb per Millionreads (RPKM). The differentially expressed genes (DEGs) are shown in *orange* and *blue*, while *brown* indicates genes that were not differentially expressed (not DEGs). **b** Venn diagram showing common or uniquely regulated genes among three samples. Upregulated genes are shown in the *left*, while downregulated genes are shown in the *right* (colour figure online)

To quantify the expression level of UP (control), SP (incompatible pollination), and CP (compatible pollination), RPKM values were calculated. A total of 1795 genes were differentially expressed between UP and SP (Additional file 1), including 896 downregulated and 899 upregulated genes. Similarly, 1890 genes were differentially expressed between UP and CP (Additional file 2), including 734 down-regulated and 1156 up-regulated genes. In addition, CP and SP were also compared: 520 genes were differentially expressed (Additional file 3), including 427 upregulated and 93 downregulated genes. The plots of unigenes between orange and blue revealed unigenes with both fold change and significance (Fig. 1a).

To further narrow the candidate genes related to incompatible and compatible reactions, DSI (UP vs. SP), DSC (UP vs. CP), and X (CP vs. SP) were comprehensively compared. As shown in Fig. 1b, 232 genes, including three common genes, were continuously upregulated and downregulated among UP, CP, and SP. Specifically, the three continuously downregulated genes were glycerophosphoryl diester phosphodiesterase (Pbr002321.1), alpha-galactosidase (Pbr002935.1), and epidermis-specific secreted glycoprotein EP1 (Pbr038211.1). Although some of these downregulated genes are not related to the two important KEGG pathways, they are important for research on pear SI.

### Gene ontology (GO) annotation and KEGG pathway mapping of all DEGs in DSI (UP vs. SP) and DSC (UP vs. CP)

**Fig. 2:**
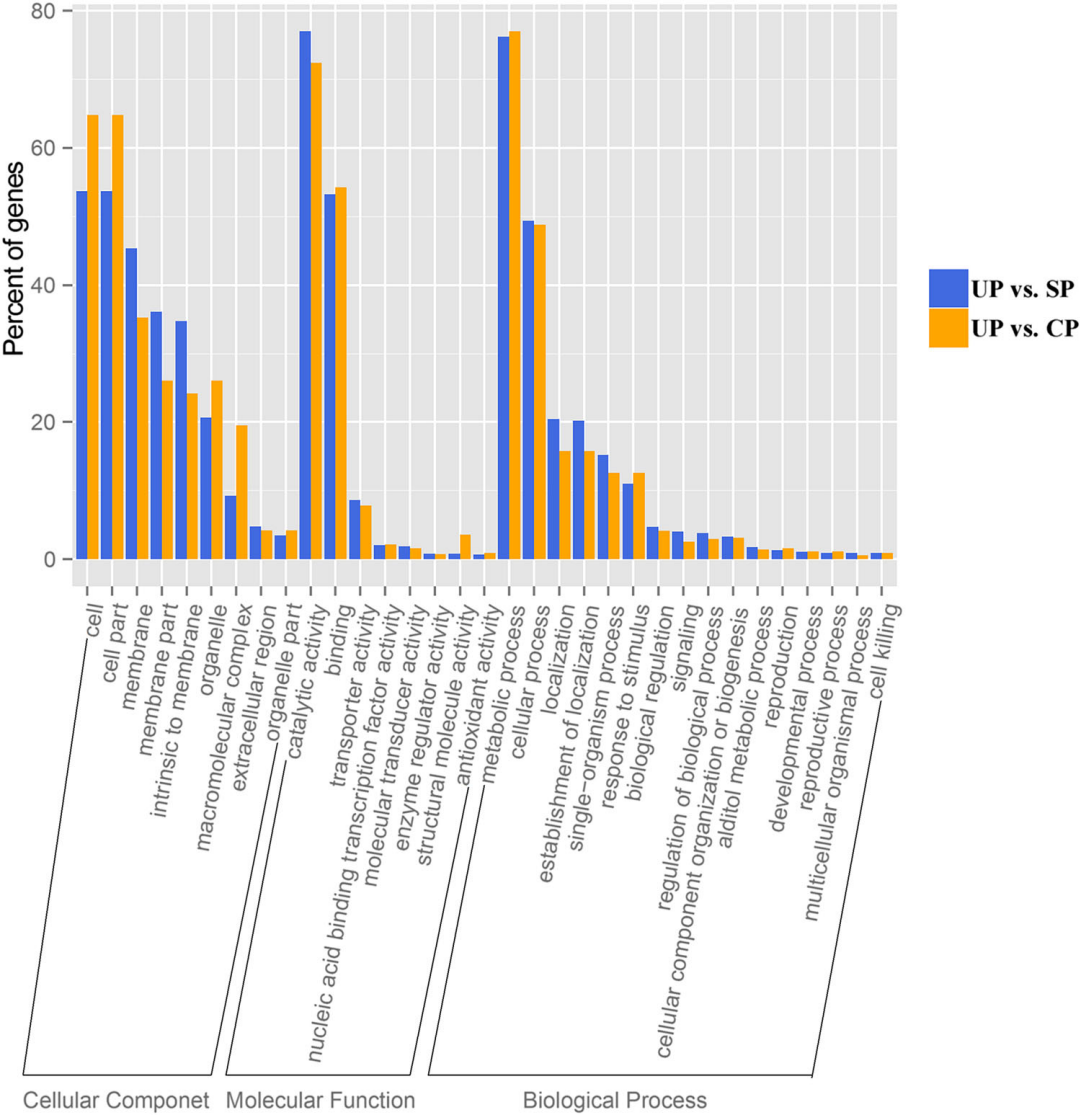
Gene ontology classification of differentially expressed genes in DSI (UP vs. SP) and DSC (UP vs. CP)

To identify the functions of DEGs in DSI and DSC, GO analyses were performed. In total, 1890 DEGs of DSI and 1796 DEGs of DSC were assigned to GO annotations. All genes were distributed into three categories: cellular component, molecular function, and biological process. The GO terms of DEGs in DSI and DSC were categorized into the same 33 main functional groups. Cell (122 genes, 53.74% in DSI; 169 genes, 64.75% in DSC), cell part (122 genes, 53.74% in DSI; 169 genes, 64.75% in DSC), and membrane (103 genes, 45.37% in DSI; 93 genes, 35.24% in DSC) dominated in the cellular component category. With respect to the molecular function, genes were associated with catalytic activity (446 genes, 77.02% in DSI; 461 genes, 72.37% in DSC), binding (308 genes, 53.19% in DSI; 346 genes, 54.31% in DSC), and transporter activity (50 genes, 0.08% in DSI; 50 genes, 0.07% in DSC). Genes associated with metabolic process (339 genes, 76.17% in DSI; 385 genes, 77.00% in DSC), cellular process (220 genes, 49.43% in DSI; 244 genes, 48.80% in DSC), and localization (91 genes, 10.44% in SI; 79 genes, 15.8% in DSC) were dominant in the biological process category (Fig. 2).

**Fig. 3:**
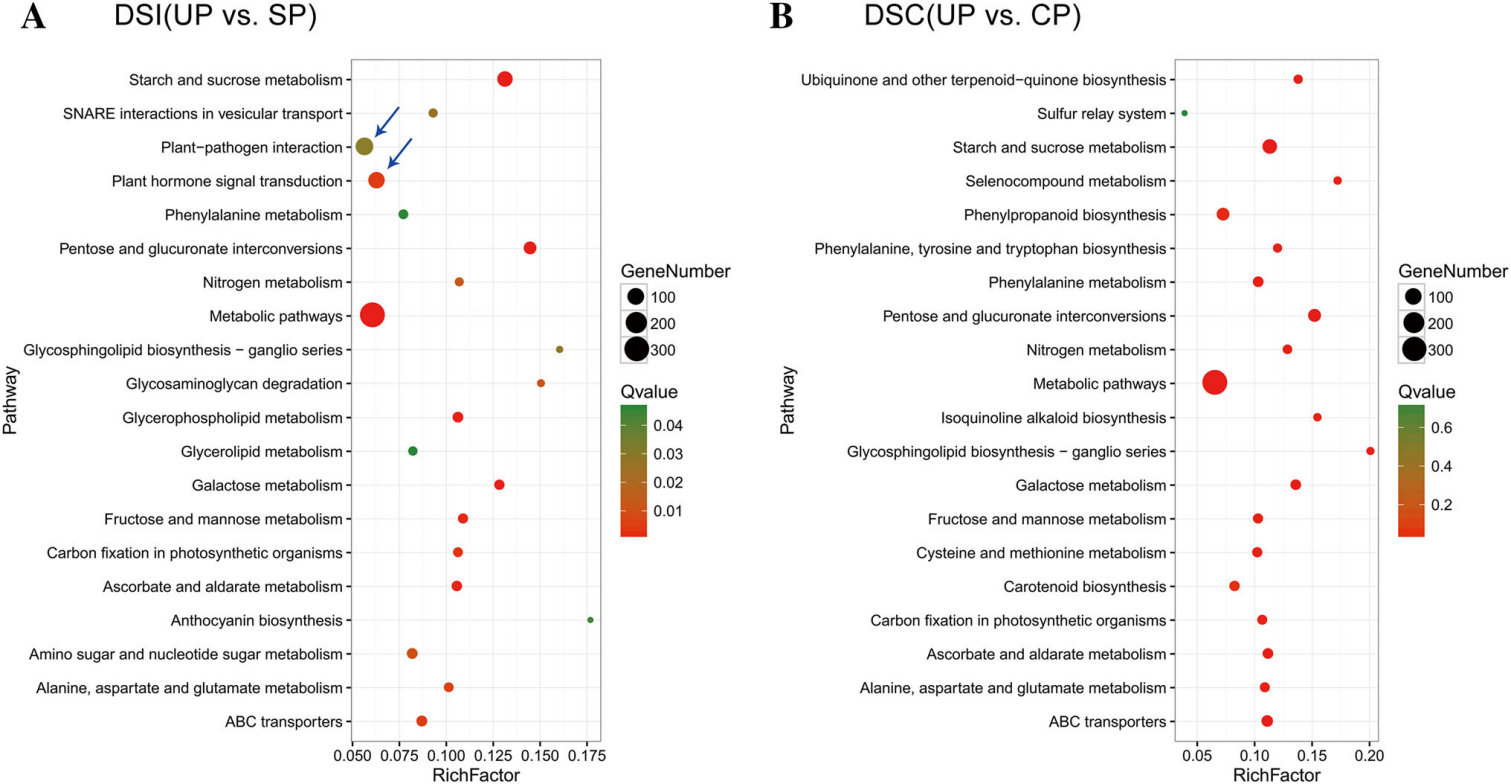
Statistical scatter diagram of KEGG pathway enrichment in DSI (UP vs. SP) and DSC (UP vs. CP). **a** Enriched pathways in DSI (UP vs. SP). *Blue arrow* shows the significantly enriched pathway between DSI and DSC. **b** Enriched pathways in DSI (UP vs. CP) (colour figure online)

To further confirm the function of DEGs in the pathways, statistical pathway enrichment analysis of DEGs in DSI (UP vs. SP) and DSC (UP vs. CP) was performed. The DSI (UP vs. SP) and DSC (UP vs. CP) DEGs were enriched in 110 and 111 KEGG metabolic pathways, respectively. The top 20 metabolic pathways of DSI and DSC, with a *P* value <0.05 and FDR <0.05, are shown in Fig. 3a. Among the 110 pathways of DSI (UP vs. SP), the three containing the highest numbers of DEGs were “metabolic pathways” (301 DEGs), “plant–pathogen interaction” (116 DEGs), and “plant hormone signal transduction” (93 DEGs). Other GO terms associated with high numbers of DEGs were “starch and sucrose metabolism” (93 DEGs), “pentose and glucuronate in interconversions” (44 DEGs), and “ABC transporters” (22 DEGs). Among the 111 pathways of DSC (UP vs. CP) (Fig. 3b), those containing the highest numbers of DEGs were “metabolic pathways” (320 DEGs), “starch and sucrose metabolism” (66 DEGs)”, pentose and glucuronate interconversions” (43 DEGs), and “ABC transporters” (28 DEGs). In addition, the DEGs in the “plant–pathogen interaction” and “plant hormone signal transduction” pathways were significant with respect to DSI vs. DSC, determined by a *P* value <0.05 and FDR <0.05.

### Expression analysis of candidate genes

**Fig. 4:**
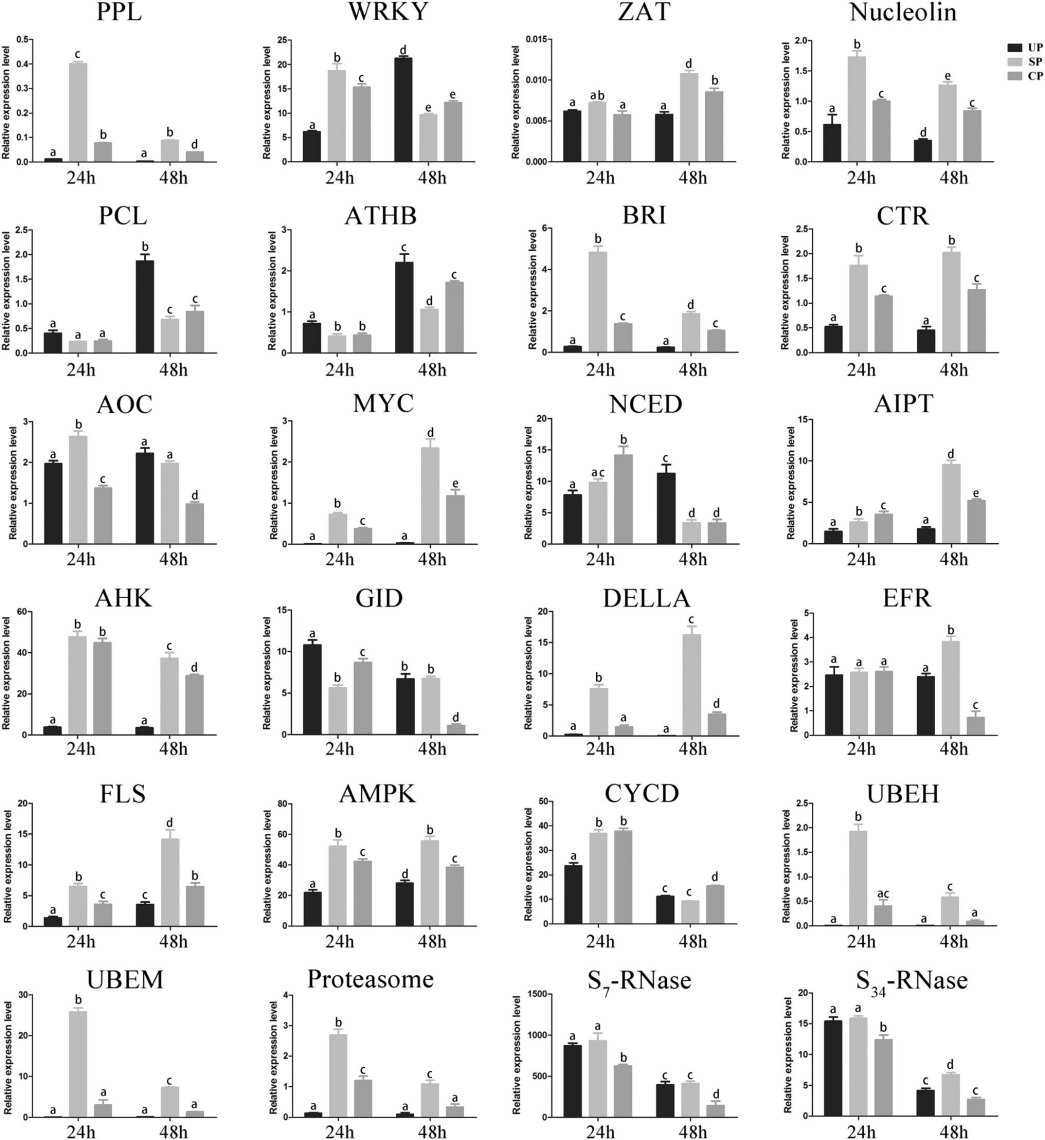
Relative gene expression of selected genes in unpollinated, self-pollinated, and cross-pollinated styles after 24 and 48 h pollination. Relative expression level was defined as the expression level and the *X-axis* indicates hours after pollination. *Bars* represent the standard deviations about the mean. *Columns labeled with different letters* are significantly different at *P* < 0.05, Duncan’s multiple range test

To evaluate the validity of RNA-seq, S-RNase and 22 candidate genes that may be associated with SI reactions were selected and evaluated by qRT-PCR using gene-specific primers (Fig. 4; Additional file 4; Additional file 5). These 22 genes were mainly related to transcription initiation, pathogen defence, and phytohormone signal transduction, including pectate lyase (PPL), transcription factor WRKY (WRKY), zinc finger protein (ZAT), nucleolin, transcription factor PCL (PCL), homeobox-leucine zipper protein ATHB (ATHB), brassinosteroid insensitive (BRI), serine/threonine-protein kinase (CTR), allene-oxide cyclase (AOC), jasmonate-associated MYC (MYC), 9-*cis*-epoxycarotenoid dioxygenase (NCED), adenylate isopentenyltransferase (AIPT), histidine kinase (AHK), GA insensitive dwarf (GID), GA sensing (DELLA), receptor-like serine/threonine-protein kinase (EFR), leucine-rich-repeat receptor kinases flagellin sensing (FLS), 5′-AMP-activated protein kinase (AMPK), Cyclin D (CYCD), ubiquitin-conjugating enzyme E2 H (UBEH), ubiquitin-conjugating enzyme E2 M (UBEM), and proteasome. Clearly, the qRT-PCR results of the selected genes showed a general agreement with the RNA-seq results (Additional file 5). Of these genes, PPL, ZAT, nucleolin, BRI, CTR, MYC, AIPT, AHK, DELLA, EFR, FLS, AMPK, UBEH, UBEM, and proteasome presented significantly higher expression levels in self-pollinated (SP) styles than in unpollinated (UP) and cross-pollinated (CP) styles at 48 h after pollination (HAP). Other genes such as WRKY, PCL, ATHB, NCED, and CYCD showed reduced levels of expression in SP styles. However, the relative expression level of CYCD was higher in CP styles at 24 HAP (Fig. 4). These results demonstrate that defence and phytohormone signal transduction were involved in the pollination reaction.

The relative expression levels of selected genes in styles at 24 HAP were also analysed (Fig. 4) and were observed difference between 24 and 48 HAP. For example, the relative expression level of PPL was higher in DSI at 24 HAP but lower at 48 HAP. The relative expression level of CYCD decreased in unpollinated, self-pollinated, and cross-pollinated styles in a time-dependent manner. In addition, the relative expression level of PCL was not affected at 24 HAP, but it was significantly increased in UP styles compared with SP/CP styles at 48 HAP.

The S-genotype of *Pyrus. bretschneideri Rehd.* cv. ‘Dangshansuli’, which was identified by PCR using sequence-conservative primers, contains two S-RNase alleles, *S*_*7*_-*RNase* and *S*_*34*_-*RNase*. Unfortunately, we failed to collect the data of *S*_*34*_-*RNase* from the RNA-seq database because of the unsuccessful assembly of S_34_-RNase in the sequenced pear genome. Consequently, two specific primer pairs for *S*_*34*_-*RNase* and *S*_*7*_-*RNase* were designed (Additional file 4), and qRT-PCR was performed to determine the relative expression levels at 24 and 48 HAP in UP, SP, and CP styles. The results showed that the relative expression levels of both *S*_*7*_-*RNase* and *S*_*34*_-*RNase* alleles were significantly lower in CP styles than in SP styles at 24 and 48 HAP (Fig. 4). These results indicate that compatible pollination could affect the expression level of S-RNase.

### Phytohormone analysis in UP, SP, and CP

**Fig. 5:**
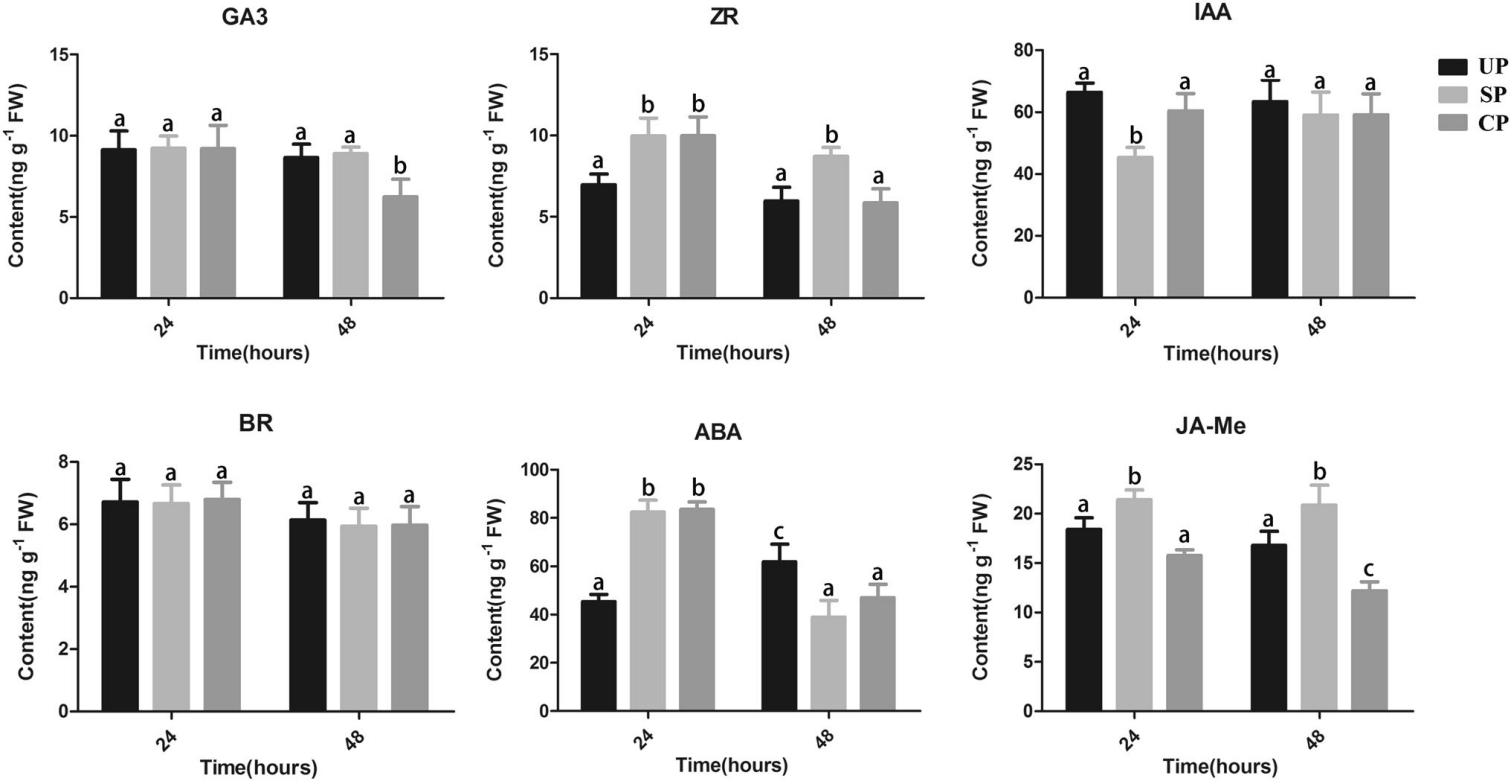
Concentration of phytohormone in UP, SP, and CP after 24 and 48 h pollination. *Bars* represent the standard deviations about the mean. *Columns labeled with different letters* are significantly different at *P* < 0.05, Duncan’s multiple range test

KEGG analysis revealed high enrichment in the plant hormone signal transduction pathway of DSI (UP vs. SP). To determine whether SI could stimulate phytohormone synthesis, UP, SP, and CP pear styles were collected at 24 and 48 HAP, and the concentrations of six endogenous phytohormones were measured. As shown in Fig. 5, at 24 HAP, GA3 and BR had almost identical concentrations among UP, SP, and CP styles. ZR and ABA had similar concentrations between SP and CP styles, which were higher than that in UP styles. IAA and JA-Me had similar concentrations between UP and CP styles, but which were higher and lower than that in SP styles, respectively. These results suggested that ZR and ABA were associated with pollen tube growth in SP and CP styles; IAA and JA-Me were only related to pollen tube growth in SP styles at 24 HAP. At 48 HAP, IAA and BR had almost identical concentrations among UP, SP, and CP styles. GA3 had similar concentrations between UP and SP styles, which were higher than that in CP styles. ZR had similar concentrations between UP and CP styles, which were lower than that in SP styles. ABA had similar concentrations between SP and CP styles, which were lower than that in UP styles. JA-Me had, respectively, higher and lower concentrations in SP and CP than that in UP styles. These results indicated that ABA was associated with pollen tube growth in SP and CP styles, ZR and JA-Me were only related to pollen tube growth in SP styles at 48 HAP. Thus, these results confirm that phytohormones play an important role in self- and cross-pollination.

### Expression level of S-RNase regulated by phytohormones

**Fig. 6:**
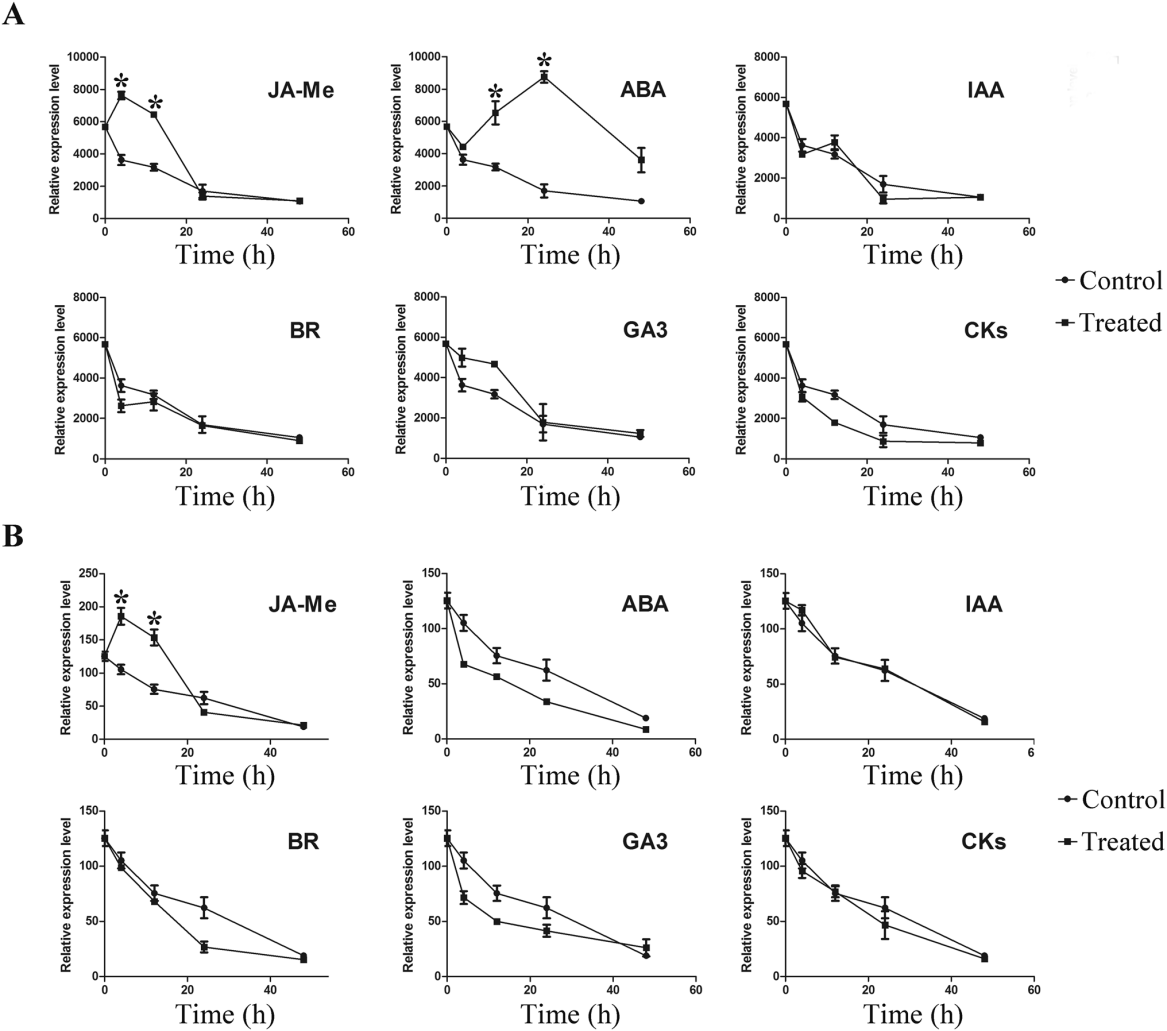
Relative expression level of S-RNase in unpollinated styles with different phytohormone treatment. **a** Relative expression level of *S*_*34*_-*RNase* treated with JA-Me, ABA, IAA, GA3, BR, and CKs. *Bars* represent the standard deviations about the mean. *Student’s *t* test with *P* < 0.05. **b** Relative expression level of *S*_*7*_-*RNase* treated with JA-Me, ABA, IAA, GA3, BR, and CKs. *Bars* represent the standard deviations about the mean. *Student’s *t* test with *P* < 0.05

RNA-seq and the phytohormone measurements demonstrate the importance of phytohormones in self- and cross-pollination. In addition, phytohormones play an important role in plant growth, development, and the response to stress. To determine whether phytohormones affect the expression level of *S*-*RNase* alleles, the UP styles were treated with JA-Me, ABA, GA3, IAA, BR, and ZR. As shown in Fig. 6a, the expression level of *S*_*34*_-*RNase* was markedly enhanced by JA-Me after 4 and 10 h. *S*_*34*_-*RNase* was also induced by ABA after 10 and 24 h. However, there was no significant difference in the expression level of *S*_*34*_-*RNase* when treated with IAA, BR, GA3, and cytokinin (zeatin). To further determine whether *S*_*7*_-*RNase* and *S*_*34*_-*RNase* have the same expression patterns, specific primers were also used to analyse the relative expression level of *S*_*7*_-*RNase*. As shown in Fig. 6b, *S*_*7*_-*RNase* was induced by JA-Me, but the ABA treatment had no effect. There is a possibility that *S*_*7*_-*RNase* and *S*_*34*_-*RNase* showed a slightly different regulatory pattern. In addition, IAA, BR, GA3, and CK could not induce the expression level of *S*_*7*_-*RNase*, which is consistent with the findings for *S*_*34*_-*RNase*.

**Fig. 7:**
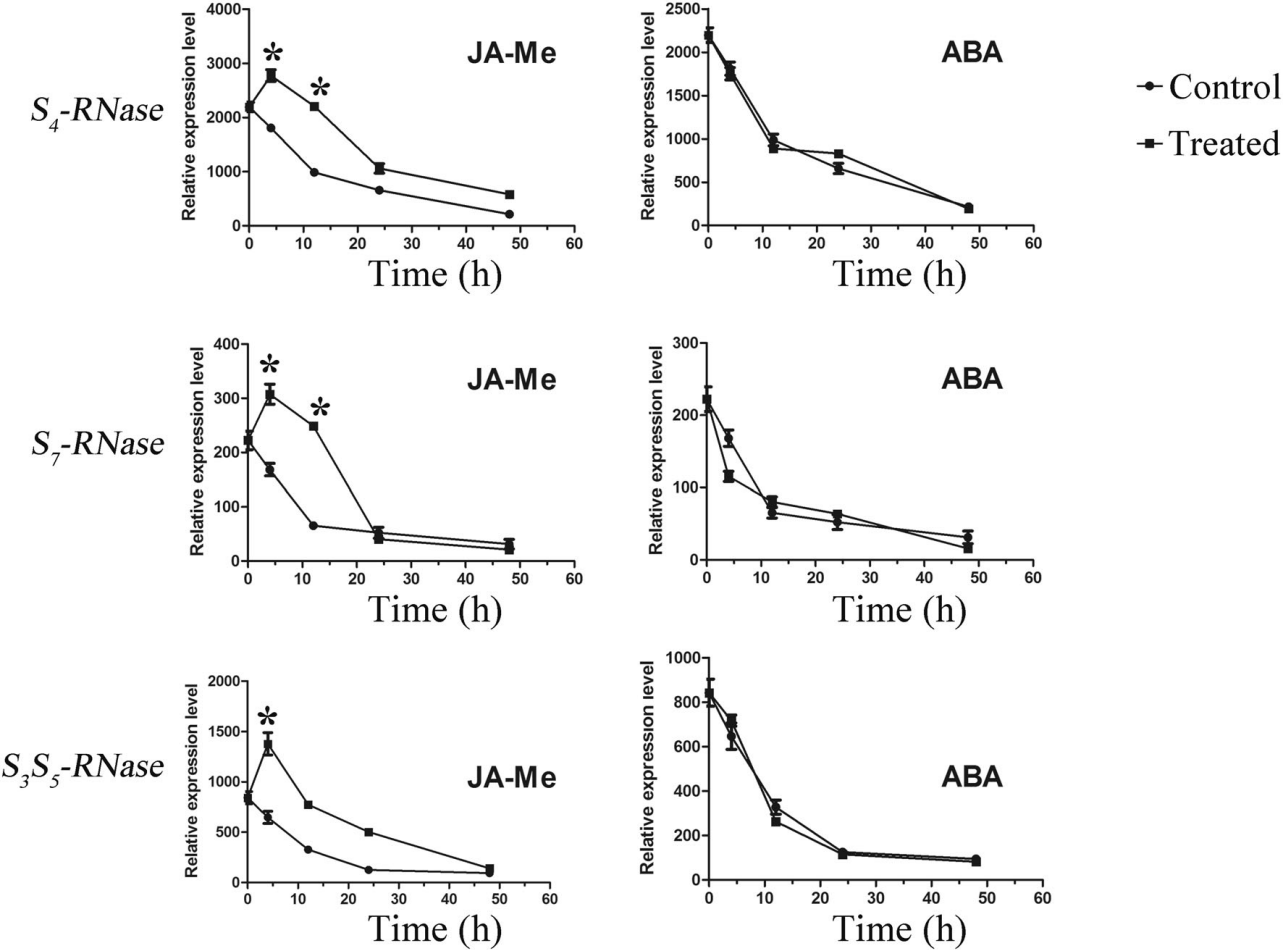
Relative expression level of S-RNase in ‘Qingxiang’ and ‘Cuiguan’ styles with JA-Me and ABA treatment. ‘Qingxiang’ harbors S4-RNase and S7-RNase; ‘Cuiguan’ harbors S3-RNase and S5-RNase

To further confirm whether JA-Me and ABA could affect the expression level of *S*-*RNase* in other styles, ‘Qingxiang’ (S_4_S_7_) and ‘Cuiguan’ (S_3_S_5_) were selected in the second year. However, specific primers were designed between *S*_*4*_-*RNase* and *S*_*7*_-*RNase* in ‘Yaqing’ but could not be designed between *S*_*3*_-*RNase* and *S*_*5*_-*RNase* in ‘Cuiguan’ due to higher sequence identity in untranslated and translated region. As shown in Fig. 7, the relative expression levels of *S*_*4*_-*RNase*, *S*_*7*_-*RNase*, and *S*_*3*_ *S*_*5*_-*RNase* were markedly enhanced by JA-Me after 4 and 10 h but could not be induced by ABA. Overall, we found a relationship between phytohormones and the expression level of S-RNase.

## Discussion

### DEGs involved in SI in pear

SI is a complex biological process that enforces outcrossing in plant populations. Exploration of the molecular mechanisms underlying the style response to self-pollen, particularly the regulation of S-RNase and the detoxification mechanism of pollen-specific S-locus *SFB*, has been important in plant genetic manipulation, genomic research, and agriculture production. RNA-seq is a rapid and efficient tool for obtaining a global profile of gene expression at the transcriptome level. Combined with the whole-genome database, it provides an improved bioinformatics pipeline to identify candidate genes involved in SI. In this study, 1795 DEGs of DSI, 1890 DEGs of DSC, and 520 DEGs of X (CP vs. SP) were identified in pear styles (Fig. 1); these genes provide further support that SI is regulated by multiple genes. This result is consistent with that observed for tomato styles, where 804 and 1341 DEGs were reported to be involved in incompatible and compatible pollination, respectively (Zhao et al. 2015). Moreover, 4785 and 7677 DEGs in lemon styles were associated with incompatible and compatible pollination, respectively (Zhang et al. 2015). In the tea SI reaction, 3182, 3575, and 3709 DEGs were identified between SP and CP styles at 24, 48, and 72 h, respectively. The expressions of these genes changed with pollen tube growth (Zhang et al. 2016). This finding suggests that SI is a complex process that requires many aspects of gene regulation. However, when comparing CP and SP, only 520 DEGs were identified, which was approximately threefold lower than DSI and DSC in our study. This result suggests that the majority of DEGs may respond to the pollination reaction, whereas fewer DEGs respond to the selection of incompatible and compatible reactions. Thus, these data demonstrate that SI is a complex process regulated in time and space.

### Involvement of phytohormones in the SI reaction

Small signalling molecules such as JA-Me are essential for plant survival in nature (Chini et al. 2007). JA-Me is a key signal in the plant response to environmental stress, such as wounding, pest attack, and pathogens (Lorenzo and Solano 2005). Our results unexpectedly indicated that the concentration of JA increased in the SP styles and decreased significantly in the CP styles (Fig. 5). In addition, JA-Me can induce the expression of S-RNase in styles. The so-called octadecanoid biosynthesis pathway is a JA biosynthesis pathway (Schaller 2001; Vick and Zimmerman 1984). Although there were no significant DEGs in DSI at 48 HAP, a key enzyme for JA biosynthesis, allene oxide cyclase (AOC, Pbr013257.1) (Li et al. 2005), was significantly downregulated in DSC but not in DSI at 48 HAP that was upregulated in DSI at 24 HAP. MYC, a basic helix–loop–helix leucine zipper transcription factor localized in the nucleus, is induced by JA. JA can induce two groups of genes: genes involved in defence responses against pathogens that are repressed by MYC and genes involved in JA-mediated systemic responses to wounding that are activated by MYC (MYC) (Lorenzo et al. 2004). We observed that the expression level of jasmonate-associated MYC (MYC, Pbr029553.1) was lower in DSC compared to that in DSI at 24 and 48 HAP, which is consistent with the reduced concentration of JA-Me in DSC. In addition, the expression level of MYC in DSI and DSC was higher at 48 HAP than at 24 HAP (Fig. 4). However, although the trend of the expression level of MYC was not consistent with a group of genes involved in defence response against pathogens in *Arabidopsis*, it was related to another group involved in JA-mediated systemic responses to wounding. This shows that signal transduction of MYC in pear may be different from that in Arabidopsis. Combined with exogenous JA treatment, we believe that the reduced expression level of S-RNase is related to the JA-signalling cascade.

ABA is an important regulator of several physiological and plant defence processes such as the response to heat and drought stresses: similar to JA, ABA can also be induced by water stress (Jubany-Marí et al. 2010; Planchet et al. 2011). The carotenoid biosynthesis pathway was enriched in DSI and DSC. Endogenous ABA biosynthesis is regulated by 9-*cis*-epoxycarotenoid dioxygenase (NCED, Pbr009089.1), which was significantly downregulated in DSI and DSC. Phytohormone analysis showed that the ABA concentration increased at 24 HAP but decreased at 48 HAP in DSI and DSC (Fig. 5). This result is consistent with the ABA synthesis pathway in 48 HAP. PP2C phosphatases, i.e., negative regulators of the ABA signal, can be induced in response to ABA, salt, and drought treatments (Chen et al. 2015). In the current study, the expression levels of PP2C phosphatases (Pbr033165.1, Pbr041497.1, Pbr022745.1, Pbr013576.1, Pbr015521.1, Pbr018965.1, Pbr032029.1, Pbr042867.1, Pbr013022.1, Pbr020818.1, and Pbr011405.1) were enriched in self- and cross-pollination. However, we could not confirm which PP2C phosphatases were the major participant in self-pollination. This is an interesting topic for our further research on pear SI. Taken together, these results suggest that the ABA-signalling cascade participates in the style response to pollen.

Cytokinin (CK) can regulate cell division, leaf senescence, nutrient mobilization, apical dominance, and seed germination (Hwang et al. 2012). In *Petunia hybrid* plants, the inhibition of pollen tube growth coincided with a fivefold increase in the CK content in the style (Kovaleva and Zakharova 2004). In the present study, the concentration of ZR increased in SP/CP styles after 24 h and decreased 48 h after cross-pollination. This result suggests that the signalling cascade of cytokinin changed when the pollen tube reached the base of the style, and the signalling cascade of cytokinin decreased at 48 h after cross-pollination. Adenylate isopentenyltransferase (AIPT, Pbr038052.1) is a key enzyme involved in zeatin biosynthesis. In addition, histidine kinase (AHK, Pbr026909.1) is a cytokinin receptor involved in cytokinin signal transduction. In our study, these two genes were significantly upregulated in DSI but not in DSC. This is consistent with the change in the concentration of ZR in the styles, suggesting that cytokinin participates in pollen discrimination but not in the synthesis of S-RNase in pear. A recent study revealed that CK is involved in a number of plant–pathogen interactions (Naseem et al. 2012). It was speculated that cytokinin participated in reducing the plant–pathogen interactions in the CP styles. Based on these findings, it can be concluded that CK is also involved in the SI reaction in pear.

The DELLA protein acts as a plant growth repressor, and GID1 is a GA receptor. GAs trigger the formation of a GA–GID1–DELLA complex, which forms ubiquitinated DELLA. Finally, DELLA is degraded by 26S protein and the plant growth repressor is dismissed. In the present study, the expression level of GA sensing (DELLA, Pbr035217.1) was enhanced in SP pear styles, which is consistent with self-pollinated lemon (Zhang et al. 2015). This finding suggests that a universal phenomenon exists in the function of DELLA in GSI. In addition, the expression level of GA insensitive dwarf (GID, Pbr029089.1) was lower in CP styles which are consistent with the reduced concentration of GA in CP styles. Taken together, it can be concluded that GA is also involved in the SI reaction in pear.

Thus, the results suggest that phytohormones are involved in the SI reaction in pear.

### Plant–pathogen signal in the SI reaction

The interaction between pollen and style shares similarities with bacterial infection in terms of biological responses (Van Doorn and Woltering 2008) and some pathogen defence genes, such as leucine-rich-repeat receptor kinases flagellin sensing (FLS) and LRR receptor-like serine/threonine-protein kinase (EFR) (Tintor et al. 2013). The increased expression of FLS (Pbr037634.1) and EFR (Pbr029003.1) in DSI suggests the activation of pathogen defence, which is similar to the SI reaction in that defence is initiated by styles to inhibit growth. By contrast, the expression levels of FLS (Pbr037634.1) and EFR (Pbr029003.1) decreased in DSC compared with DSI. Styles can recognize self-/cross-pollen, just as plants can discriminate between wounding and pathogen invasion. It is possible that the change in the expression level of S-RNase is a signal in pollen recognition. These results suggest that some kind of signal is required to reduce the S-RNase level (Liu et al. 2009).

The pathogen invasion response was highly enriched in the “plant–pathogen interaction” KEGG pathway (*P* value <0.05), which involved PAMP-triggered immunity, a hypersensitive response, phytoalexin accumulation, and other responses to fungus in DSI but not in DSC (Fig. 3a). In tomato, although pollination induces the same KEGG pathway, there was no significant difference between compatible and incompatible pollination at 24 HAP (Zhao et al. 2015). In addition, it was speculated that SI has evolved from defence against pathogens (Kao and McCubbin 1996), and the defence reaction was weak at 24 HAP because of the expression levels of FLS and EFR mentioned above. It is possible that the pollination time leads to a different KEGG pathway enrichment. We assume that incompatible and compatible pollen tubes were recognized as foreign incursions at the beginning of pollination. With the continuous pollen-style communication, the style recognizes incompatible pollen as a foreign incursion and triggers the defence reaction; however, compatible pollen will be accepted and the defence reaction will decrease. We concluded that plant–pathogen interactions participate in the pear SI reaction and in the pollen discrimination in pear.

### Style S-RNase biosynthesis

S-RNase is a major protein that is involved pollen recognition and is expressed mainly in styles. A direct method to inhibit SI in pear is to knock out the S-RNase gene. Because of a longer period of early development and late flowering stage and genetic diversity (Wu et al. 2013), it is difficult and time-consuming to produce self-compatible transgenic breeds. Another indirect method to inhibit SI is to regulate the S-RNase expression or translation levels in real time. During the young bud stages, the SI reaction is weak in some GSI species (Gradziel and Robinson 1989; Qin et al. 2006). Although young bud pollinations are frequently used to overcome SI in certain species (Gradziel and Robinson 1989), they are not widely used in agriculture production largely due to petal detachment. In the present study, the reduced expression level of S-RNase in compatible pollination was consistent with the reduced concentration of JA-Me in the CP styles. In addition, S-RNase in the UP styles decreased at the translation level, and the expression level decreased in a time-dependent manner after flowering (Fig. 6). It seems that a compatible pollen tube can reduce S-RNase, and a mechanism exists in the style that degrades S-RNase at the translation level, reducing the expression level in cross-pollination at post flowering. Therefore, further studies are required to design methods to accelerate this process, which would help in overcoming SI in agriculture production.

S-like RNases can be induced in response to mechanical wounding and phytohormone treatment (Groß et al. 2004; Rogers and Rogers 1999; Hillwig et al. 2008). In the present study, JA-Me induced the expression of S-RNase, but the concentration of JA-Me in self-pollinated styles was not significantly higher than that in UP styles. In addition, the concentration of JA-Me was lower in CP styles, which could be a contrary evidence to prove that a relationship exists between JA-Me and the expression of *S*-*RNase*. We suggest that JA-Me is involved in pear SI via the induction of the expression of S-RNase. However, more work is required to identify the downstream factors. ABA could only induce *S*_*34*_-*RNase*, and the effect of ABA was delayed (Fig. 6). ABA triggered JA accumulation in *Salvia miltiorrhiza* hairy roots (Yang et al. 2012), but further studies are required to confirm whether it can trigger JA biosynthesis in pear styles. The ABA signals appear to share different mechanisms with JA in regulating the expression of S-RNase.

### Potential interference of the DEG results

Because it is difficult to remove the pollen tube from SP to CP styles, we selected styles containing pollen. Hence, there is the possibility of pollen interference in the few DEGs, or genes were not significantly expressed in the styles. Nevertheless, the quantity of pollen in the styles was relatively lower than the total quantity of the styles, and research (Zhang et al. 2015, 2016; Zhao et al. 2015) on SI in other species confirmed our finding that styles containing pollen can be used to analyse the SI reaction in styles. In addition, the expression level of some organ-specific genes such as style-specific S-RNase was not affected. Thus, although there was a potential interference of the DEG results, this method can help in narrowing the range of candidate genes in further research on the SI reaction.

In summary, a comprehensive transcriptome-based characterization of the DEGs involved in self- and cross-pollination was performed in pear styles. Candidate genes related to incompatibility and compatibility reactions were identified. Two major KEGG pathways, “plant hormone signal transduction” and “plant–pathogen interaction”, were emphasized in pear SI reaction. JA-Me and ABA may enhance the expression level of S-RNase and pollination could affect the synthesis of JA-Me and ABA in pear styles. It appeared that there is relationship between self-rejection, plant hormones, and pathogen defence in pear SI. Therefore, the question of how phytohormones affect the synthesis of S-RNase, how pathogen defence participates in pear SI, can be examined further.

#### **Author contribution statement**

DS carried out the experiments, data analysis, and preparation of figures, and drafted the manuscript. CT and RW participated in the experiments and data analysis. CG participated in the data analysis and preparation of figures, and contributed with consultation. XW, SH, and JJ contributed to sample collection and data analysis. SZ managed and designed the research and experiments.

## Electronic supplementary material

Below is the link to the electronic supplementary material.

Supplementary material 1 (XLS 527 kb) Additional file 1. RNA-seq data of all counts for DSI (UP vs. SP)

Supplementary material 2 (XLS 774 kb) Additional file 2. RNA-seq data of all counts for and DSC (UP vs. CP)

Supplementary material 3 (XLS 149 kb) Additional file 3. RNA-seq data of all counts for and X (CP vs. SP)

Supplementary material 4 (DOC 39 kb) Additional file 4: table. S1 Primers used for RT-qPCR analysis of the genes

Supplementary material 5 (TIFF 1086 kb) Additional file 5: Figure. S1 The qRT-PCR validation of selected differential genes detected via RNA-Seq after 48 h pollination. Columns show the results of RNA-seq; the line charts show the results of qRT-PCR validation

## Abbreviations

24 HAP: 24 Hours after pollination
48 HAP: 48 Hours after pollination
AMPK: 5′-AMP-activated protein kinase
NCED: 9-*cis*-epoxycarotenoid dioxygenase
AOC: Allene-oxide cyclase
AIPT: Adenylate isopentenyltransferase
ABA: Abscisic acid
BR: Brassinolide
BRI: Brassinosteroid insensitive
CK: Cytokinin
CYCD: Cyclin D
CP: Cross-pollinated
DEGs: Differentially expressed genes
UP vs. CP: DSC
UP vs. SP: DSI
FDR: False discovery rate
GSI: Gametophytic SI
GA3: Gibberellins
GID: GA insensitive dwarf
DELLA: GA sensing
AHK: Histidine kinase
ATHB: Homeobox-leucine zipper protein ATHB
RNA-seq: High-throughput Illumina RNA sequencing
IAA: Indole-3-acetic acid
MYC: Jamonate associated MYC
KEGG: Kyoto Encyclopedia of Genes and Genomes
FLS: Leucine-rich-repeat receptor kinases flagellin sensing
JA-Me: Methyl jasmonate
PPL: Pectate lyase
PCD: Programmed cell death
EFR: Receptor-like serine/threonine-protein kinase
GABA: γ-Amino butyric acid
SP: Self-pollinated
CTR: Serine/threonine-protein kinase
S-RNase: S-glycoprotein ribonuclease
SSI: Sporophytic SI
SI: Self-incompatibility
WRKY: Transcription factor WRKY
PCL: Transcription factor PCL
TTS: Transmitting tissue-specific proteins
UP: Unpollinated
UBEH: Ubiquitin-conjugating enzyme E2 H
UBEM: Ubiquitin-conjugating enzyme E2 M
CP vs. SP: X
ZAT: Zinc finger protein
ZR: Zeatin riboside
