# Supplementary material for: Transcriptome and phytohormone analysis reveals a comprehensive phytohormone and pathogen defence response in pear self-/cross-pollination
Source: Plant Cell Rep. 2017 Sep 8;36(11):1785–99. doi: 10.1007/s00299-017-2194-0 (PMC5658469; doi:10.1007/s00299-017-2194-0)
Supplement: Supplementary file 4 — Supplementary material 4 (DOC 39 kb) Additional file 4: table. S1 Primers used for RT-qPCR analysis of the genes [file 299_2017_2194_MOESM4_ESM.doc]

Table S1 Primers used for RT-qPCR analysis of the genes

| Gene name | Gene ID | Sequence（5'-3')-forward | Sequence（5'-3')-reverse |
| --- | --- | --- | --- |
| PPL | Pbr039133.1 | TGGCATTACCCTCCAGTTTG | CATCACATCATCGTGGTCAGTG |
| WRKY | Pbr034115.1 | ACAACCATCCCAAGCCACAG | CCCTCCTCCTGACTTGCTCC |
| ZAT | Pbr005045.1 | AGTCACAAGAAACCTGGTAAG | ATGCCCTGAAAGCCCTATT |
| Nucleolin | Pbr004401.2 | GGCGAGTAGTAGCGTTGCG | CTGCTGCTGCCTCCATTTC |
| PCL | Pbr018943.1 | CGGTGGAGACGATGGAGAC | CGGAATCAACGGCTGAGATA |
| ATHB | Pbr017259.1 | CACTTGTACTCGGCCTCTGA | GCTTCTCCACCTTTGCTCTT |
| BRI | Pbr011934.4 | TGCCATCGCTGTCATCGTAG | CCCTTCTTTCCACTCCCTCTG |
| CTR | Pbr014132.1 | CACTATAAGAGCAGGAGGGAG | CGTCGGGGTTCAGAGTAAGGA |
| AOC | Pbr013257.1 | CATTTCTCCAACTCCATCAGG | TTGAACTTTTGTGGGTCGTG |
| MYC | Pbr029553.1 | TGGTTCCTCCTCGGCTATG | AAGTGGCTTTGATGGGTGC |
| NCED | Pbr009089.1 | GCAGCTACAGGTACACGGGTTA | GGATGTTGGTAGGTAGGGGAGT |
| AIPT | Pbr038052.1 | ATTCACAAGGGACAAGGAGG | TAGCCAGATGGCTTGAGCT |
| AHK | Pbr026909.1 | CACGACGGCGGCGTTAGTA | GCACTGTGGCTTTGACTTTGTC |
| GID | Pbr029089.1 | CCCAAATCTCCCTCAAAACC | TGAAGAGCAGCCCAGCAGT |
| DELLA | Pbr035217.1 | GGACCAAAACTCTTACTCCCTG | GGAGGTCACTTCTATCGCAAC |
| EFR | Pbr029003.1 | TGGGTGCATACCTCCCTCAA | GTGGCATTGGTCAAGTCCGA |
| FLS | Pbr037634.1 | GAGACACCTCCCAGACACCA | GCAGTTGAGACGAGCATCG |
| AMPK | Pbr035731.1 | GGTCGTGCGGCGTGATAC | CGGGTCGCTGGGTTTGTAT |
| CYCD | Pbr022031.1 | CGGAGGAGCATTTGGTGG | AGGGGCTGATTTGGAGGG |
| UBEH | Pbr041262.1 | GAGGGTGAGATCCCGAAAAG | CGTCCAAGGAGGAAGACCAGT |
| UBEM | Pbr028213.1 | TCTACCCTCACGAGCCTCCG | GGTCCTCATGGTTAGGTTCCGT |
| proteasome | Pbr016136.1 | GCAGCGAGATCAGCAACGA | TCAGGAGCGGCATCAGGTG |
| Dangshan-S7-RNase | Pbr002004.1 | CAGCTGTCTGCAACTCCAAAC | GTCGTTCATTATTGCGGGAT |
| Dangshan-S34-RNase |  | AACATGGTACCTGTGGAGTC | CTGCGTATGGCTTTTAGAAT |
| Qingxiang-S4-RNase |  | TTCAACCGAACGGGAATAAC | GTGATCCTTTTGGAGGCCC |
| Qingxiang-S7-RNase |  | AGATTGAACCGTTGGGAATAC | ATCCTGGTGGTGGGTGGG |
| Cuiguan-S3S4-RNase |  | CTCAAGTGCCAAAAGAAGGG | ATTGGTTCAAAGGGGTGGG |
| actin | Pbr035825.1 | TACTCTTTCACCACAACTGC | CTCGTAACTCTTCTCCACAG |
