# Supplementary figures and images for: Transcriptome and phytohormone analysis reveals a comprehensive phytohormone and pathogen defence response in pear self-/cross-pollination
Source: Plant Cell Rep. 2017 Sep 8;36(11):1785–99. doi: 10.1007/s00299-017-2194-0 (PMC5658469; doi:10.1007/s00299-017-2194-0)

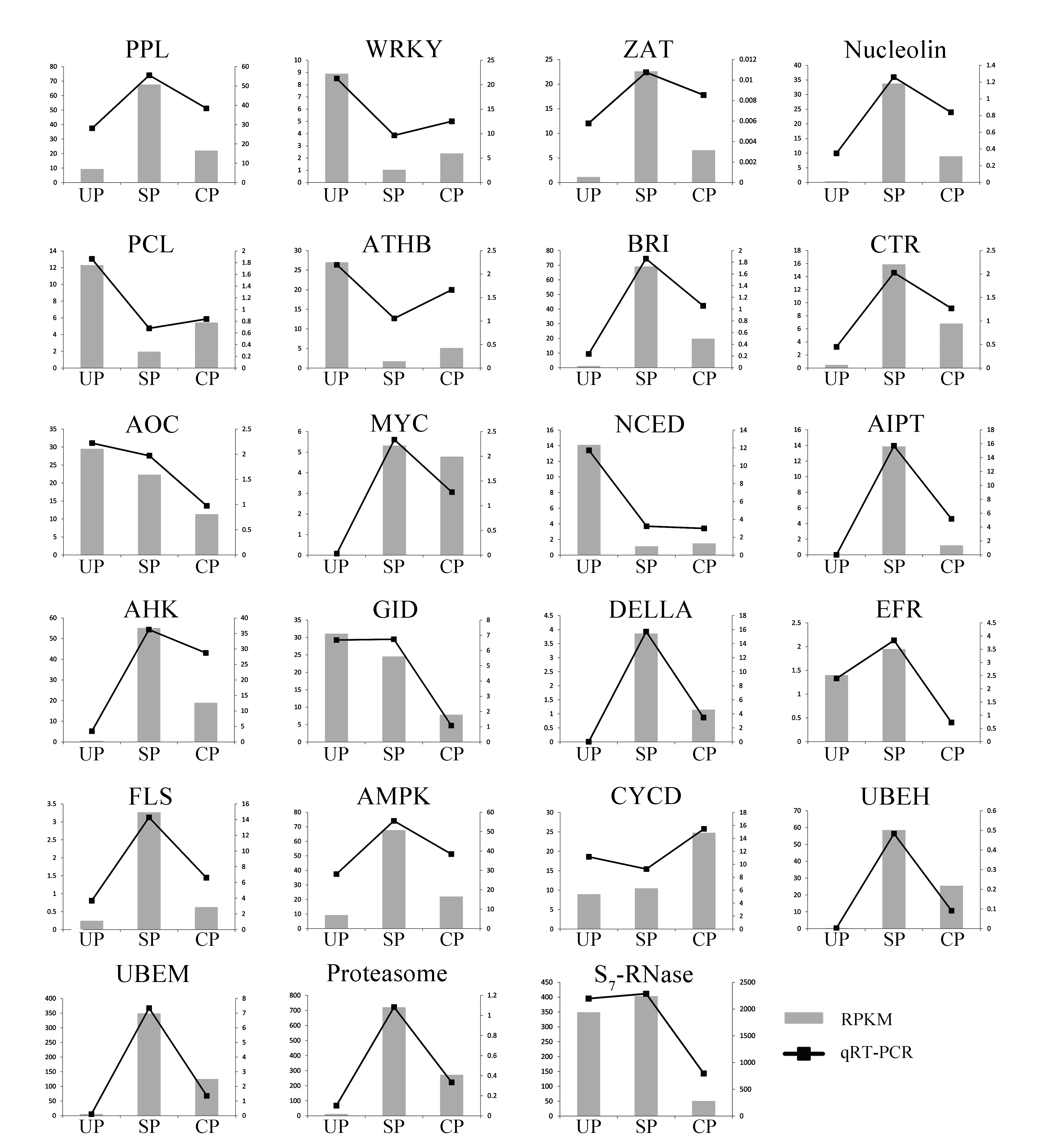

Supplement: Supplementary file 5 — Supplementary material 5 (TIFF 1086 kb) Additional file 5: Figure. S1 The qRT-PCR validation of selected differential genes detected via RNA-Seq after 48 h pollination. Columns show the results of RNA-seq; the line charts show the results of qRT-PCR validation [file 299_2017_2194_MOESM5_ESM.tif]
